# Effects of Employee Well-Being and Self-Efficacy on the Relationship between Coaching Leadership and Knowledge Sharing Intention: A Study of UK and US Employees

**DOI:** 10.3390/ijerph182010638

**Published:** 2021-10-11

**Authors:** Wenxian Wang, Seung-Wan Kang, Suk Bong Choi

**Affiliations:** 1 College of Business, Gachon University, Seongnam 13120, Korea; wangwenxian@gachon.ac.kr; 2 College of Global Business, Korea University, 2511 Sejong-ro, Sejong City 30019, Korea

**Keywords:** coaching leadership, employee well-being, knowledge sharing intention, self-efficacy, conservation of resources theory

## Abstract

Knowledge acquisition practices are important to enterprises, particularly since market competition is intensifying. In recent years, organizations have begun to pay more attention to knowledge sharing practices. Many organizations are looking for methods to motivate their employees to actively share knowledge with other employees. This study uses the conservation of resources theory to examine coaching leadership as an antecedent—and employee well-being as a mediator—in facilitating knowledge sharing intention; it finds that self-efficacy is the boundary condition in these relations. We collected data in two waves and recruited participants online—full-time employees in the UK and US. Using a sample of 322 employees, we conducted a confirmatory factor analysis to test the validity of the results and used hierarchical multiple regression to examine the direct and interaction effects. Then, we used the bootstrapping method to test the indirect and moderated mediation effects. Our results show that coaching leadership is positively related to knowledge sharing intention, and employee well-being mediates the relationship. Moreover, self-efficacy positively moderates the direct and indirect effects. Our findings demonstrate that employee well-being is a mediating mechanism in the relationship between coaching leadership and knowledge sharing intention, with self-efficacy acting as a boundary condition.

## 1. Introduction

Since the advent of the knowledge economy era, competition among enterprises has become fiercer. The literature demonstrates that knowledge sharing is associated with organizational innovation [1] and is key to organizational success [2]. Knowledge updates are more frequent, and knowledge sharing is essential for the sustainable development of enterprises. Through the promotion of knowledge sharing, an organization enhances and maintains its core competitiveness [3]. Knowledge sharing is a voluntary act, and it is unlikely that employees will automatically share their valuable knowledge with others [4]. Managers need to motivate employees to share knowledge actively to gain competitive advantages [5]. According to Maier [6], knowledge is an employee’s resource. Personal knowledge and expertise affect the employee’s status and interests in an organization [7]. Unless employees are willing to share their knowledge, an organization cannot achieve the goal of knowledge sharing. Knowledge transfer to the outside world must be based on a certain willingness. Therefore, this study focuses on knowledge sharing intention.

Knowledge is a source of power and advantage [8]. Employees who possess ‘unique knowledge’ often experience positive performance evaluations, personal gains, and protection from layoffs [9]. Thus, it is a competitive advantage for employees [10]. Due to the complex environments of organizations and the scarcity of knowledge resources [11], employees may be reluctant to share their knowledge.

Knowledge sharing is challenging and requires time and effort [12], which reduces the competitive advantages of knowledge providers [7] and the resources unique to knowledge providers [13]. According to the conservation of resources (COR) theory [14], when individuals have more resources, they are less affected by the loss of resources. Therefore, individuals always attempt to obtain more resources [15]. According to Schaufeli and Bakker [16], employees’ access to relevant resources can increase their voluntary behavior in their workplace. Therefore, it is necessary to provide sufficient resources to compensate for the risks involved in individual knowledge sharing [7,17]; otherwise, employees may not have knowledge sharing intentions.

Previous studies noted that leaders can affect the degree of knowledge sharing [4,18] at the team level. The mechanism through which leaders influence knowledge sharing at the individual level has not been explored [19]. Unlike other leadership styles that focus on the interests of an organization, coaching leadership focuses on improving employee skills, providing resources, enhancing their competitiveness, supporting employees to achieve their goals [20,21,22], and providing resources to employees. Thus, we use coaching leadership as an antecedent of knowledge sharing intention.

According to the COR theory, resource loss is associated with stress and burnout, and the pressure-induced work environment discourages employees from engaging in civic behaviors, such as knowledge sharing [23]. Coaching leadership can provide resources, reduce employee stress and burnout, improve employee well-being, and promote employee knowledge sharing behavior. Therefore, this study uses employee well-being as the mediating role between coaching leadership and knowledge sharing intention based on the COR theory.

Additionally, according to the resource caravan passageway principle of the COR theory [24], different resources interact with each other. In the workplace, work resources are the factors that initiate the motivational process, such as the support supervisors provide to help employees meet their basic needs. However, personal resources refer to an individual’s own perceptions of his/her ability to successfully control and influence the environment [25]. Self-efficacy is an important personal resource [26] that reflects an individual’s perceptions of his/her ability to conduct a specific behavior [27], and according to the COR theory, work and personal resources interact with each other to influence outcomes. Wang and Noe [17] proposed that the interaction of individual differences and situational factors can help predict whether an individual is likely to share knowledge with others, and Constant et al. [28] found that individuals with higher specialized knowledge, i.e., self-efficacy, were more likely to share useful knowledge. Therefore, in this study, we employed self-efficacy as a key variable to moderate the indirect relationship between coaching leadership and knowledge sharing intention through employee well-being.

**Figure 1:**
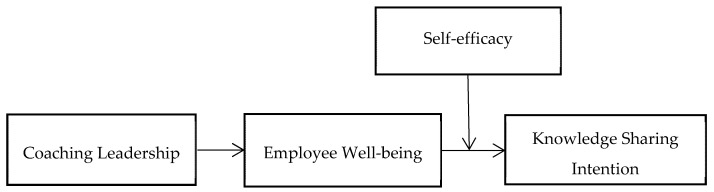
Hypothesized research model.

In general, we find that coaching leadership is the antecedent of knowledge sharing intention and it contributes to the growing leadership literature. This research clarifies how coaching leadership affects knowledge sharing intention. The following paragraphs will list the basic assumptions of the COR theory and relate coaching leadership to knowledge sharing intention. We also focus on the mediating role of employee well-being and examine self-efficacy as a moderating variable. Then, we describe the research methods and results, discuss this research’s theoretical contributions and practical applications, and provide suggestions for future research. Figure 1 depicts the hypothesized research model.

## 2. Theoretical Background and Hypotheses

### 2.1. Coaching Leadership and Employee Well-Being

Hamlin, Ellinger, and Beattie [21] defined coaching leadership as a leadership style that helps employees acquire new skills, improve their capabilities and performance, and enhance their efficiency, growth, and development. Hagen [22] defined coaching leadership as a leadership style that can help improve the employee’s performance through job performance management, as well as improve the employee–manager relationship. Cardoso and Ramos [29] divided coaching leadership into several structural elements: communication, giving and receiving feedback, authorization, influence, and supporting the team to achieve organizational goals.

Diener [30] defined well-being as a comprehensive assessment of people’s feelings and attitudes about their lives. Warr [31] defined employee well-being as a qualitative assessment of employees’ feelings and functioning at work. Grant et al. [32] defined employee well-being as the happiness that employees derive from their work, including satisfaction with their work’s intrinsic and extrinsic value. Employee well-being is always associated with the leadership style in an organization [33].

The COR theory suggests that individuals seek to acquire resources to prevent resource loss. The potential threat of or actual resource loss is related to individual tension and stress burnout [14,24]. Resources can be goals, conditions, states, and anything else people value [14,15]. The more resources individuals have, the more likely they are to be free from experiencing psychological stress [34], whereas fewer resources trigger more tension and stress. Because coaching leadership can provide resources for subordinates, we hypothesize that coaching leadership has an impact on employee well-being.

During work, employees deplete their resources over time. Halbesleben et al. [15] argued that resource loss at work is more harmful to employees than resource gain, and it can explain employees’ job stress and well-being due to resource loss. Individuals avoid future resource losses in the form of lower well-being [35]. The support from supervisors is a key mechanism for employees to replenish their resources, so organizations can maintain and acquire resources by providing opportunities to employees [36]. Coaching leadership provides guidance, helps subordinates achieve their work goals, helps superiors form high-quality relationships with subordinates, and provides employees with many work resources that can directly foster positive emotions and reduce negative emotions and stress, thus enhancing employee well-being. Therefore, we hypothesize that coaching leadership has a positive relationship with employee well-being.

The extant research shows that employee perception of supervisor support is related to employee well-being [37]. Skakon [38] indicated that a leader’s behavior is associated with employee well-being. Coaching leadership provides support and helps employees to be successful [39]. Coaching leadership helps followers perceive that the interaction and relationship with their leader are types of support and resources, affecting their well-being [15]. Coaching leaders support their followers to grow and provide them with useful resources. Moreover, coaching leadership is valuable and effective in reducing and coping with stress and enhancing employee well-being [40]. Thus, coaching leadership can influence employee well-being, so we propose the following hypothesis:

**Hypothesis** **1** **(H1).** *Coaching leadership positively relates to employee well-being.*

### 2.2. The Mediating Role of Employee Well-Being in the Relationship between Coaching Leadership and Knowledge Sharing Intention

Bock, Zumd, Kim, and Lee [41] defined knowledge sharing intention as the extent to which employees are willing to share knowledge. Knowledge sharing refers to providing expertise to others and collaborating with others to solve problems, developing new ideas, or implementing procedures [17]. Knowledge sharing behavior is not spontaneous, and knowledge sharing intention plays a decisive role in it [42]. The knowledge sharing intention of employees significantly predicts the actual knowledge sharing behavior in an organization [43].

According to the COR theory, insufficient resources to cope with work-related demands cause stress and burnout [44]. When there are fewer resources or a loss of resources—stress and burnout occur. Those with fewer resources are motivated to avoid further loss of resources and, thus, avoid investing in additional resources [14]. The COR theory suggests that individuals strive to acquire, maintain, nurture, and protect the resources they value. Individuals continually invest in resources to protect the existing resources from loss and acquire new resources [34]. When people get enough resources, stress, and burnout reduce, employee well-being increases, and they tend to behave positively. Moreover, to avoid the loss of existing resources, people will invest in additional resources. Knowledge sharing, which benefits organizational managers, can be regarded as a type of resource investment, which prevents a future loss of employee resources. Therefore, we speculate that coaching leadership has indirect effects on knowledge sharing intention through employee well-being.

People are motivated to protect their resources [14] because knowledge sharing is challenging and consumes the knowledge provider’s resources [13]. Coaching leadership provides employees with work guidance, helps them achieve their work goals, provides them with work resources, reduces their pressure, and promotes a high-quality working environment, thereby improving their employee well-being. Moreover, it helps employees to acquire resources. Employees with more resources have a stronger intention to take challenges and engage in knowledge sharing.

After obtaining such resources, employees will try to maintain, cultivate, and protect their cherished resources. To avoid possible resources loss in the future, employees will invest resources and expect reciprocal resources from supervisors or colleagues. Knowledge sharing can help the development of an organization, which will benefit the supervisors. When employees invest in resources through knowledge sharing, they expect to acquire resources from their supervisors in a reciprocal way to avoid possible resource losses in the future. Coaching leadership improves employees’ resources and nourishes their well-being. To protect their well-being, employees will invest their resources in their organization by sharing their knowledge with others to benefit their leaders to avoid possible future loss of resources. Therefore, we propose that employee well-being mediates the indirect effect of coaching leadership on knowledge sharing intention.

Ellinger et al. [45] suggested that supervisory coaching behavior is positively related to performance. Kim [39] proved that managerial coaching has an indirect impact on job performance through role clarity. Hagen and Aguilar [46] indicated that coaching expertise explains the variance in team learning outcomes. Connelly and Kelloway [47] found that management’s support affects knowledge sharing. The positive relationship between perceived organizational support and knowledge sharing intention has been proven [48]. 

Wasko and Faraj [49] indicated that individuals are willing to share knowledge when they experience enjoyment and happiness. Moreover, knowledge sharing leads to better performance evaluation and career development opportunities [50,51], and it is a good resource investment. Employee well-being induces more employee extra-role behaviors, such as knowledge sharing, to promote the organization’s development. Therefore, we propose the following hypothesis:

**Hypothesis** **2** **(H2).** *Employee well-being mediates the relationship between coaching leadership and knowledge sharing intention. Coaching leadership enhances employee well-being, and the increased employee well-being leads to higher knowledge sharing intention.*

### 2.3. The Moderating Role of Self-Efficacy on the Relationship between Employee Well-Being and Knowledge Sharing Intention 

Self-efficacy can be seen as competence [52], a person’s belief that they can perform activities skillfully [53]. Bandura [54] defined self-efficacy as an individual’s belief in their ability to produce a given level of performance. Deci and Ryan [55] defined self-efficacy as a simple conceptualization of behavioral competence. Individuals bring traits related to self-efficacy into the workplace [56]. Bandura [27] contends that self-efficacy is different from confidence in that self-efficacy is about perceived competence in a particular behavior, whereas confidence lacks definite goals and that self-efficacy represents an affirmation of the capability and power of that belief, whereas confidence only reflects the strength of certainty about a performance or perception, additionally, self-efficacy is based on theory and considerable empirical data, whereas confidence is often used without a theoretical basis.

The COR theory predicts that resources have synergistic effects [14]. The principle of resource caravans and resource caravan passageways explicitly states that different resources are not independent but have interconnections and influences (similar to a traveling caravan) [24]. This principle means that resources may interact with each other and influence an outcome. According to the COR theory’s initial resource effect corollary [15], individuals with more initial resources are less likely to suffer from resource loss. They have a relatively greater ability to acquire new resources. Individuals with fewer initial resources are more likely to suffer from resource loss and have a relatively weaker ability to acquire new resources. Moreover, as an individual resource, self-efficacy affects the relationship between employee well-being and knowledge sharing intention.

Self-efficacy is a kind of personal resource [26] that helps individuals retain resources, gain additional resources (e.g., optimism), or better withstand stressful conditions [24]. Employees with high self-efficacy believe that they have high levels of competence, have more resources, and are less likely to lose resources. Thus, they are more likely to set higher work goals and job roles and share their knowledge at work to help the organization grow. Conversely, employees with low self-efficacy have fewer resources, are more likely to suffer from resource loss, and prefer to reduce their work effort to conserve resources. Therefore, we hypothesize that employees with high self-efficacy have higher knowledge sharing intention, whereas those with low self-efficacy have lower knowledge sharing intention.

Bock and Kim [42] found that self-efficacy leads to more positive sharing attitudes. Wenger & Snyder [57] proposed that knowledge sharing should be encouraged by promoting participants’ sense of self-efficacy, helping them to build expertise, and providing recognition. In summary, self-efficacy affects the relationship between employee well-being and knowledge sharing intention; thus, we propose the following hypothesis:

**Hypothesis** **3** **(H3).** *Self-efficacy moderates the relationship between employee well-being and knowledge sharing intention such that the relationship is stronger for employees with higher self-efficacy.*

### 2.4. The Moderated Mediating Role of Self-Efficacy

The COR theory of resource gain spirals corollary [15] states that initial resource gain leads to further resource gain, and individuals in the process of acquiring resources are less stressed and more advantaged in resource investment. Conversely, the resource loss spirals corollary [15] states that initial resource loss triggers further loss of resources. Because resource loss triggers tension and stress, individuals will have fewer resources to stop resource loss and have difficulty developing effective resource investments. Thus, it becomes difficult to develop effective resource investments to prevent resource loss. As a resource, self-efficacy can moderate the indirect effect of coaching leadership on knowledge sharing intention through employee well-being.

Sharing knowledge with colleagues can threaten their performance by increasing competition [7], anxiety, and stress. Employees who have self-efficacy are more likely to have more positive relationships with others [58]. Positive personal and external resources facilitate knowledge sharing [59]. Individuals with high self-efficacy are more capable of interacting and collaborating at work, building high-quality relational resources with leaders, and having higher levels of well-being. People with high self-efficacy tend to evaluate potential stressful situations as challenges rather than threats, which is conducive to coping with stress [26].

Self-efficacy can effectively demonstrate personal characteristics, knowledge, skills, professional attitudes, and values to provide safe and effective output [60]. Employees with high self-efficacy are more willing to overcome the challenges of knowledge sharing and invest resources to respond to the additional expectations of their leaders [61,62,63]. However, individuals with low self-efficacy are not competent in work, leading to work pressure, a low sense of happiness, and difficulty developing additional resource investment. They are not willing to make resource investments and have no intention to conduct knowledge sharing to avoid loss. Therefore, we hypothesize that the indirect effect of coaching leadership on knowledge sharing intention through employee well-being is more positive when self-efficacy is high than when it is low.

Self-efficacy is feeling effective in ongoing interactions with the social environment and experiencing opportunities to exercise and master one’s abilities [55]. High self-efficacy is positively associated with well-being [64]. Self-efficacy positively impacts an employee’s performance at work because it increases expectations for him at work, leading to higher demands on his role [65]. Self-efficacy enables employees to handle new and difficult tasks, which will lead to excellent work results and company success [66]. When subordinates have greater self-efficacy, they expect to contribute to task performance [67]. 

Locke and Schweiger [68] and Locke, Feren, McCaleb, Shaw, and Denny [69] considered subordinates’ self-efficacy a moderating variable in the relationship between participation in decision-making and job performance. Kim and Jung [70] indicated that self-efficacy could be a buffer against the negative effects of work stress on employees’ safety behavior. Peng et al. [71] argued that self-efficacy plays a moderating role in social relationships and knowledge sharing. To explain how self-efficacy affect how coaching leadership facilitates knowledge sharing intention, we propose a moderated mediation model in Figure 1 and the following hypothesis:

**Hypothesis** **4** **(H4).** *Self-efficacy moderates the indirect effect of coaching leadership on knowledge sharing intention through employee well-being such that the indirect relationship is stronger for employees with higher self-efficacy.*

## 3. Method

### 3.1. Sample 

We recruited full-time employee participants working in UK and US from an online panel platform—Prolific Academic. This online panel platform is designed for academic research [72] and is as reliable [73], and has the same quality as conventional field samples [74]. To reduce common method bias (CMB), we employed a multi-time approach for the data collection and embedded attention checks in each wave [75,76]. We conducted two separate waves with separate questionnaires. Following previous research about well-being [77], we conducted a two-wave survey separated by six weeks.

In the first wave, the participants measured their perception of their leader’s coaching leadership. In the second wave, the participants measured their employee well-being, self-efficacy, and knowledge sharing intention. In the first wave, we received 422 responses, and 376 responses remained after we excluded incomplete surveys. In the second wave, we sent questionnaires to the 376 qualified participants from the first wave and received 326 responses. After we excluded incomplete surveys, 322 completed questionnaires remained as our final sample.

In the sample, 44.4% are males, and 55.6% are female. Regarding nationality, 78.26% of the respondents are from the UK, and 21.74% are from the US. Respondents with high school education are 15.83%; 19.25% have college diplomas; 43.18% have bachelor’s degrees; 17.39% have master’s degrees, and 4.35% have Ph.D. The average age is 38.92 years (SD = 9.93), and the mean of organization tenure is 7.86 years (SD = 6.99). The mean interaction frequency with the supervisor is 21.35 times in a week (SD = 23.73). 

### 3.2. Measures

#### 3.2.1. Coaching Leadership 

Coaching leadership was measured using a four-item scale employed by Farh and Chen [78]. An example of the items on the scale is “My supervisor provides explanations and discusses the procedure, diagnostics, equipment, and supplies.” We measured these items on a five-point Likert scale ranging from 1 (strongly disagree) to 5 (strongly agree). The Cronbach’s alpha is 0.89 (see Appendix A for a complete list of the items).

#### 3.2.2. Employee Well-Being

Employee well-being was measured using a four-item scale developed by Brunetto, Farr-Wharton, and Shacklock [79]. An example of the items on the scale is “Overall, I think I am reasonably satisfied with my work life”. We measured these items on a five-point Likert scale ranging from 1 (strongly disagree) to 5 (strongly agree). The Cronbach’s alpha is 0.85 (see Appendix A for a complete list of the items).

#### 3.2.3. Self-Efficacy

We employed a three-item scale developed by Spreitzer [52] to measure employees’ self-efficacy. An example of the items on the scale is “I am confident about my ability to do my job”. We measured these responses on a five-point Likert scale ranging from 1 (strongly disagree) to 5 (strongly agree). The Cronbach’s alpha is 0.86 (see Appendix A for a complete list of the items).

#### 3.2.4. Knowledge Sharing Intention 

We used a self-report three-item scale adapted from the study of Huang, Davison, and Gu [80] to measure my knowledge sharing intention [81]. An example of the items on the scale is “I intend to share knowledge with my colleagues when they ask”. We measured these items on a five-point Likert scale ranging from 1 (strongly disagree) to 5 (strongly agree). The Cronbach’s alpha is 0.88 (see Appendix A for a complete list of the items).

#### 3.2.5. Control Variables 

We included employee age, gender, education, organization tenure [82], and nationality as control variables to account for demographic differences, influencing knowledge sharing intention. Age and organization tenure were measured in years. Gender was coded as a dichotomous variable—0 for females and 1 for males. Nationality was also coded as a dichotomous variable—0 for United Kingdom and 1 for United States. Education was divided into six levels—elementary school, high school, college diploma, bachelor’s degree, master’s degree, and Ph.D. 

Howell et al. [83] suggested that interaction frequency influences employee performance. We included interaction frequency as a control variable. It is measured in numbers by the question, “How many times do you interact with your supervisor in a week, including talking to him or her in person, on the phone, and by email?”

### 3.3. CMB

To reduce CMB, we used a multi-time approach for the data collection. However, because all the variables were measured using responses from the same participants, it will result in false internal consistency, potentially leading to misleading results [84]. We conducted Harman’s single-factor test to assess the effects of common method variance [85]. We loaded all the items of the measured construct onto the exploratory factor analysis, and the covariance among the variables shows that no single-factor explained more than 40% of the variance [86]. Based on the analysis result, we determined that the CMB of this study did not significantly influence the validity of the results. 

### 3.4. Analytical Strategy

The analysis of all the variables in the study was conducted at the individual level with STATA 15.1 (Data Analysis and Statistical Software, Stata Corp., College Station, TX, USA). First, we conducted a confirmatory factor analysis to test the distinctiveness of the variables. Then, we conducted the chi-square model comparison test. We used hierarchical multiple regression analyses to test the effects. We mean-centered the independent and moderating variables to create the product term to reduce the collinearity between the composite constructs of the product term. We used the bootstrapping method to examine the indirect effect of coaching leadership on knowledge sharing intention [87]. Moreover, we used the bootstrapping resamples method to analyze the moderated mediation proposed in H4.

## 4. Results 

### 4.1. Descriptive Statistics 

Table 1 presents the Cronbach’s alpha, means, standard deviation, and correlations of the variables. The Cronbach’s alpha of variables are between 0.85 and 0.89, which show good reliability. Coaching leadership is significantly correlated with employee well-being (r = 0.49; *p* < 0.001) and knowledge sharing intention (r = 0.21; *p* < 0.001). Moreover, multicollinearity is not a serious problem in our analyses because, in the regression analyses, all the variance inflation factors of the independent variables are below 10 [88].

### 4.2. Confirmatory Factor Analysis and Chi-Square Difference Test

The model fit indices are the comparative fit index (CFI) and Tucker–Lewis index (TLI), which are ≥0.90, and the root mean square error of approximation (RMSEA) is ≤0.08 [89]. The hypothesized research model—a four-factor model—has sufficient fit indices (χ² = 215.69; df = 71; CFI = 0.95; TLI = 0.93; RMSEA = 0.08). Compared with other alternative models, the four-factor model is the best, as shown in Table 2.

### 4.3. Hypotheses Tests 

H1 predicted that coaching leadership positively relates to employee well-being. After controlling gender, age, education, organization tenure, nationality, and interaction frequency, Table 3 shows a significant positive relationship between coaching leadership and employee well-being (β = 0.47; *p* < 0.001; Model 2). The analytical results support H1.

H2 predicted that employee well-being mediates the relationship between coaching leadership and knowledge sharing intention. Coaching leadership enhances employee well-being, and the increased employee well-being leads to higher knowledge sharing intention. We used 10,000 replications of bootstrapping; the observed coefficient effect is 0.10, and the 95% bias-corrected bootstrap confidence interval is (0.06, 0.14), which does not contain zero. The mediator variable, employee well-being, is related to the dependent variable, knowledge sharing intention (β = 0.17; *p* < 0.001; Model 6). The analytical results support H2.

**Figure 2:**
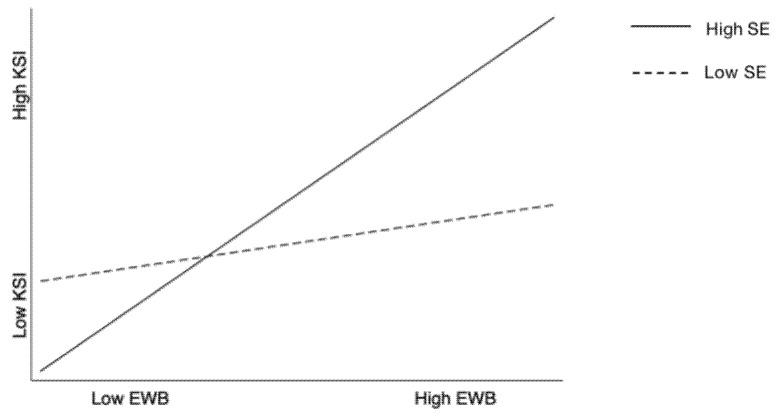
The moderating effect of self-efficacy on the relationship between employee well-being and knowledge sharing intention. EWB = employee well-being SE = self-efficacy KSI = knowledge sharing intention.

H3 proposes that self-efficacy moderates the relationship between employee well-being and knowledge sharing intention such that the relationship is stronger for employees with higher self-efficacy. The interaction coefficient between employee well-being and self-efficacy is significantly positive (β = 0.16; *p* < 0.001; Model 6). The simple slope in Figure 2 shows that employee well-being is positively related to knowledge sharing intention when self-efficacy is high (simple slope = 0.30; SE = 0.05; *p* < 0.001), but not significant when self-efficacy is low (simple slope = 0.07; SE = 0.05; ns). The analytical results support H3.

H4 proposes that self-efficacy moderates the indirect effect of coaching leadership on knowledge sharing intention through employee well-being. The indirect relationship is stronger for employees with higher self-efficacy. With a 95% bias-corrected confidence interval, the bootstrapped resample results show self-efficacy moderates the positive indirect effect of coaching leadership on knowledge sharing intention through employee well-being. The moderated mediation effect is positive when self-efficacy is high (indirect effect = 0.14; SE = 0.03; BCa CI = [0.09, 0.21]), but the moderated mediation effect is not supported when self-efficacy is low (indirect effect = 0.02; SE = 0.03; BCa CI = (−0.03, 0.08)). The results of the analysis support H4.

## 5. Discussion

As business competition becomes increasingly fierce, employee knowledge sharing within an organization is crucial to the competitiveness of the organization [3]. Employees’ knowledge sharing intention directly impacts employees’ knowledge sharing behavior [42,43]. As the major influencers in the workplace, leaders can influence the increase or decrease in personal resources and thus influence employees’ knowledge sharing intention. Based on the COR theory, this study investigated the effect of coaching leadership on knowledge sharing intention and the mediating role of employee well-being in the relationship between coaching leadership and knowledge sharing intention. It found that when employees’ self-efficacy is high, the effect of coaching leadership on knowledge sharing intention is enhanced. Conversely, when the self-efficacy level is low, the effect of coaching leadership on knowledge sharing intention is not significant.

### 5.1. Theoretical Implications 

First, our study confirms the results of previous literature. Some studies have indicated that leaders’ behavior may be a key factor that promotes or hinders employees’ knowledge sharing behavior [4]. Some studies have also shown that management support positively affects employees’ knowledge sharing behavior [17]. In our study, coaching leadership was selected as an ideal resource provider that positively affects knowledge sharing intention and, thus, promotes knowledge sharing. 

Second, our study describes the mechanism of how coaching leadership affects the knowledge sharing intention of employees. Our results indicate that employee well-being plays a mediating role in the relationship between coaching leadership and employee knowledge sharing intention. The existing literature proves that the relationship between perceived organizational support and knowledge sharing intention is mediated by affective organizational commitment [48]. Our study uses the COR theory to find that employee well-being is the psychological mechanism that mediates the relationship between coaching leadership and knowledge sharing intention. Coaching leadership provides resources to improve employee well-being. Employees who want to protect resources and avoid resources loss in the future invest in resources and engage in knowledge sharing to benefit their leaders and organization.

Moreover, only a few studies have paid attention to the boundary conditions of coaching leadership. The existing studies about the boundary conditions of coaching leadership often focus on proactive personality [90]. Our study examines self-efficacy as a potential moderator of the relationship between coaching leadership and knowledge sharing intention. First, we hypothesize that self-efficacy moderates the relationship between employee well-being and knowledge sharing intention such that the relationship is significant only when self-efficacy is high. Second, we hypothesize that the indirect relationship between coaching leadership and knowledge sharing intention through employee well-being depends on self-efficacy. The indirect relationship will be significant only when self-efficacy is high. In previous studies, self-efficacy is often used as the antecedent of knowledge sharing intention, and research on self-efficacy as the boundary effect is limited. Therefore, this study enriches the knowledge sharing literature by exploring the boundary role of how self-efficacy affects knowledge sharing intention.

Finally, our study extends the COR theory. Wu and Lee [91] used the COR theory to examine how abusive leadership influences psychological capital and knowledge sharing with the moderating role of group trust. However, our study, which applies the COR theory, reveals how coaching leadership provides resources to subordinates through guidance, reduces employees’ stress and burnout, improves employee well-being, and enhances employees’ knowledge sharing intention. 

### 5.2. Managerial Implications

Our study has several implications for organizations and managers. First, a leader’s behavior is an important factor that affects employees’ knowledge sharing intention. Therefore, managers should pay attention to employees, help their development, and improve their well-being by investing in employees to promote their knowledge sharing intention. Moreover, this study advises organizations to recruit and develop leaders with coaching tendencies of listening, helping, supporting development, and empowering rather than pursuing power. In addition, organizations should invest more time developing and providing leadership training, such as providing leadership training courses and imparting interpersonal skills to supervisors. These investments will promote employee well-being and enhance their knowledge sharing intention.

Moreover, our research suggests that organizations should place more emphasis on strengthening employees’ self-efficacy. We suggest that managers pay more attention to employees’ self-efficacy to increase their knowledge sharing intention in the organization. Moreover, they can devise recruitment processes to screen employees with high levels of self-efficacy. Managers should also pay attention to cultivating employees’ self-efficacy and training employees to improve their self-efficacy. The human resource department also needs to use various strategies to explore employees’ self-efficacy to promote their career development [92]. Increased self-efficacy can enhance employees’ knowledge sharing intention and promote the organization’s development.

### 5.3. Limitations and Future Studies

Our study has several limitations that could be explored in future research. First, the variables, coaching leadership, self-efficacy, employee well-being, and knowledge sharing intention, were measured using self-report responses from the same participants. Although Chan [93] indicated that self-report does not influence data correlation, it may increase CMB. Spector [94] highlighted that CMB is not the main issue with the validity of the results. We still use a multi-time approach by separating the two waves by six weeks. We conducted Harman’s one-factor test to examine the effect of CMB on our results, which is not adequate and limits the causality of the research. Future research can employ the multisource approach.

Second, we researched the individual levels of perception of coaching leadership, but leadership is a ground-level construct related to its members [95]. Individual differences influence the interpretation and response to supervisor behavior [96,97]. Future research can analyze the team level or multi-level.

Third, we screened participants for United Kingdom and United States nationality so that our sample would comprise native speakers, but this limits the generalizability of these findings. In the future, we could translate our questionnaires to other languages and extend our study to other countries.

Fourth, we did not conduct comparative analysis between the UK and US participants in this study, but future researchers could compare respondents from different countries to ascertain differences. 

Fifth, some scholars have proposed that employee well-being is not stable and changes with time [98,99]. However, our study does not consider the changes in employee well-being with time. Therefore, a future study should use a longitudinal design to observe the changes in employee well-being over time.

Sixth, some studies suggest that some other leadership styles, such as transformation and ethical leadership, influence employee well-being [100]. Therefore, future research can use other leadership styles that can affect employee well-being as the control variable.

## 6. Conclusions

In this study, we used the COR theory to examine coaching leadership as an ideal resource that positively affects employee knowledge sharing intention and reinforces employee well-being as a mediator in the relationship between coaching leadership and knowledge sharing intention. We also highlighted the role of self-efficacy as a boundary condition in the direct effects between employee well-being and knowledge sharing intention and in indirect effects between coaching leadership and knowledge sharing intention. Specifically, we found that the direct and indirect effects were both significant under high self-efficacy, but not low. Furthermore, we extended the COR theory by highlighting coaching leadership as a resource that enhances employee well-being and thereby promotes employees’ knowledge sharing intention. Although there are some limitations of our study, these findings contribute to the growing research on coaching leadership and knowledge sharing intention.

## Figures and Tables

**Table 1:** Cronbach’s alpha, means, standard deviations, correlations, and reliabilities.

Variable	Alpha	Mean	SD	1	2	3	4	5	6	7	8	9	10
1. Gender (0 = F; 1 = M) ^a^		0.44	0.50	-									
2. Age		38.92	9.93	0.03	-								
3. Education		3.75	1.06	0.03	−0.07	-							
4. Organization tenure ^b^		7.86	6.99	0.05	0.47 ***	−0.15 **	-						
5. Nationality		0.22	0.41	0.10	0.06	0.04	−0.10	-					
6. Interaction frequency ^c^		21.35	23.73	0.01	0.02	−0.17 **	0.02	−0.06	-				
7. Coaching Leadership	0.89	3.22	0.92	0.06	−0.02	0.05	−0.04	0.10	0.14 *	-			
8. Self-efficacy	0.86	4.21	0.72	−0.05	0.17 *	−0.08	0.10	0.13 *	0.10	0.13 *	-		
9. Employee Well-being	0.85	3.44	0.92	0.02	0.08	0.13 *	−0.04	0.08	0.09	0.49 ***	0.32 ***	-	
10. Knowledge Sharing Intention	0.88	4.38	0.59	0.01	0.01	−0.03	0.01	0.07	0.16 *	0.21 ***	0.27 ***	0.35 ***	-

Note: n = 322; * = *p* < 0.05; ** = *p* < 0.01; *** = *p* < 0.001 (two-tailed); ^a^ F = female; M = male; ^b^ tenure = number of years; ^c^ frequency = times in a week.

**Table 2:** Model fit statistics for the measurement models.

Measurement Model	χ²	Df	CFI	TLI	RMSEA	Δχ²	Δdf
Baseline (hypothesized) four-factor model	215.69	71	0.95	0.93	0.08		
Alternative 1 (three-factor model) ¹	679.79	74	0.78	0.73	0.16	464.1 ***	3
Alternative 2 (two-factor model) ²	1172.79	76	0.61	0.53	0.21	957.1 ***	5
Alternative 3 (one-factor model) ³	1660.38	77	0.43	0.33	0.25	1444.69 ***	6

Note: *n* = 322. * = *p* < 0.05; ** = *p* < 0.01; *** = *p* < 0.001 (two-tailed test). ¹ A three-factor model with employee well-being and knowledge sharing intention on the same factor. ² A two-factor model with employee well-being, knowledge sharing intention, and self-efficacy. ³ A one-factor model with coaching leadership, employee well-being, knowledge sharing intention, and self-efficacy.

**Table 3:** Hierarchical multiple regression of employee well-being and knowledge sharing intention.

Variables	Employee Well-Being		Knowledge Sharing Intention			
	Model 1	Model 2	Model 3	Model 4	Model 5	Model 6
Controls						
Gender	0.01	−0.04	0.00	−0.01	0.01	−0.01
Age	0.01	0.01 *	−0.00	−0.00	−0.00	−0.00
Education	0.12 *	0.10 *	−0.00	−0.01	−0.02	−0.03
Organization tenure	−0.01	−0.01	0.00	0.00	0.00	0.00
Nationality	0.16	0.05	0.12	0.09	0.05	0.00
Interaction frequency	0.00 *	0.00	0.00 **	0.00 *	0.00 *	0.00
Independent Variable						
Coaching Leadership		0.47 ***		0.12 ***	0.02	0.03
Self-efficacy					0.13 **	0.19 ***
Interaction						
Employee Well-being*Self-efficacy						0.16 ***
Mediator						
Employee Well-being					0.18 ***	0.17 ***
Model Fit						
F	2.58 *	16.06 ***	1.67	3.11 **	6.97 ***	7.94 ***
R^2^	0.05	0.26	0.03	0.06	0.17	0.20
△F		92.46 ***		11.42 ***	19.23 ***	14.04 ***
△R^2^		0.21		0.03	0.11	0.03

Note: *n* = 322; * = *p* < 0.05; ** = *p* < 0.01; *** = *p* < 0.001 (two-tailed test). The results of the standardized regression coefficients.

## Appendix A

Coaching leadership (α = 0.89) [78]

1. My supervisor provides instruction and feedback directed toward mastery.
2. My supervisor updates status for the task objective, team, environment, progress of procedure.
3. My supervisor makes sense of unexpected events, and discusses implications for the team.
4. My supervisor provides explanations and discussions about the procedure, diagnostics, equipment, and supplies.

Employee well-being (α = 0.85) [79]

1. Overall, I think being a current job worker fulfils an important purpose in my work life.
2. Overall, I get enough time in this job to reflect on what I do at work.
3. Overall I think I am reasonably satisfied with my work life.
4. Overall, most days I feel a sense of accomplishment in what I do in working.

Self-efficacy (α = 0.86) [52]

1. I am confident about my ability to do my job.
2. I am self-assured about my capabilities to perform my work.
3. I have mastered the skills necessary for my job.

Knowledge sharing intention (α = 0.88) [80]

1. I will make an effort to share knowledge with my colleagues.
2. I intend to share knowledge with my colleagues when they ask.
3. I will share knowledge with my colleagues.
